# 

**Published:** 1880-10

**Authors:** 


					﻿SURG GENLS~OFFS
Vol. XVI.
October, 1880.
No. 3.
S E®
I fell tf lijWl I
EfeSljjTL ¡Lm. bb	g T. v% F «a/» rîtfÆ "pÌ*
\j -KHLtcff	k~T*iT fT-W?* «jjyjSSjFEr"	Tg^iï
4T» W ub	CBr mID> vt4Weh lÿ® 1JÙ
g 0 ! TQB »
ELMIRANY
				

